# PneusPage: A WEB-BASED TOOL for the analysis of Whole-Genome Sequencing Data of Streptococcus pneumonia

**DOI:** 10.71150/jm.2409020

**Published:** 2025-01-24

**Authors:** Eunju Hong, Youngjin Shin, Hyunseong Kim, Woo Young Cho, Woo-Hyun Song, Seung-Hyun Jung, Minho Lee

**Affiliations:** 1 Department of Life Science, Dongguk University-Seoul, Goyang 10326, Republic of Korea; 2 Basic Medical Science Facilitation Program, Catholic Medical Center, The Catholic University of Korea, Seoul 06591, Republic of Korea; 3 ConnectaGen, Hanam 12918, Republic of Korea; 4 Department of Biochemistry, College of Medicine, The Catholic University of Korea, Seoul 06591, Republic of Korea; 5 Integrated Research Center for Genomic Polymorphism, College of Medicine, The Catholic University of Korea, Seoul 06591, Republic of Korea

**Keywords:** *Streptococcus pneumoniae*, antimicrobial resistance, mobile genetic element, virulence factor, whole-genome sequencing

## Abstract

With the advent of whole-genome sequencing, opportunities to investigate the population structure, transmission patterns, antimicrobial resistance profiles, and virulence determinants of *Streptococcus pneumoniae* at high resolution have been increasingly expanding. Consequently, a user-friendly bioinformatics tool is needed to automate the analysis of *Streptococcus pneumoniae* whole-genome sequencing data, summarize clinically relevant genomic features, and further guide treatment options. Here, we developed PneusPage, a web-based tool that integrates functions for species prediction, molecular typing, drug resistance determination, and data visualization of *Streptococcus pneumoniae*. To evaluate the performance of PneusPage, we analyzed 80 pneumococcal genomes with different serotypes from the Global Pneumococcal Sequencing Project and compared the results with those from another platform, PathogenWatch. We observed a high concordance between the two platforms in terms of serotypes (100% concordance rate), multilocus sequence typing (100% concordance rate), penicillin-binding protein typing (88.8% concordance rate), and the Global Pneumococcal Sequencing Clusters (98.8% concordance rate). In addition, PneusPage offers integrated analysis functions for the detection of virulence and mobile genetic elements that are not provided by previous platforms. By automating the analysis pipeline, PneusPage makes whole-genome sequencing data more accessible to non-specialist users, including microbiologists, epidemiologists, and clinicians, thereby enhancing the utility of whole-genome sequencing in both research and clinical settings. PneusPage is available at https://pneuspage.minholee.net/.

## Introduction

*Streptococcus pneumoniae* (pneumococcus), a major human pathogen, is responsible for significant global morbidity and mortality, particularly in children the elderly, and immunocompromised individuals (Weiser et al., 2018; Wyllie et al., 2016). It is the causative agent of various diseases, ranging from mild respiratory infections to life-threatening conditions, such as pneumonia, meningitis, and sepsis (Loughran et al., 2019; Weiser et al., 2018; Zivich et al., 2018). The genetic diversity of *S. pneumoniae*, characterized by its numerous serotypes and the presence of drug-resistant strains, poses a significant challenge for effective clinical management and epidemiological surveillance (Chang et al., 2018; Cremers et al., 2019; Hudspeth et al., 2001; Jacques et al., 2023)

Traditionally, analyses regarding *S. pneumoniae* have relied on culture-based and molecular typing-based techniques. For example, the Quellung test and phenotypic drug susceptibility testing (DST) have been used as gold standards for serotyping and determining antibiotic resistance, respectively (Bard and Lee, 2018; Habib et al., 2014). Additionally, multilocus sequence typing (MLST) through PCR amplification has been employed to characterize bacterial strains based on the sequences of internal fragments of seven housekeeping genes (Larsen et al., 2012). While all these techniques provide valuable insights, they are time-consuming, labor-intensive, require skilled personnel, and may lack the resolution needed for differentiating closely related strains (Varghese et al., 2017).

In contrast, whole-genome sequencing (WGS) has emerged as a powerful tool for understanding the genomic landscape of *S. pneumoniae*. It enables researchers to investigate its population structure, transmission patterns, antimicrobial resistance profiles, and virulence determinants at a level of resolution unachievable with traditional typing methods (Fani et al., 2011; Li et al., 2016; Lo et al., 2022; Yan et al., 2021; Zeng et al., 2023). Through WGS analysis of *S. pneumoniae*, Lo et al. (2022) identified the Global Pneumococcal Sequence Cluster (GPSC) 10 lineage as a major driver of the increase in serotype 24F in France. This GPSC10 was found to be multidrug-resistant and had a high potential for invasive disease regardless of serotype, highlighting the challenge it poses for serotype-based vaccine strategies. Similarly, Zeng et al. (2023) investigated the evolutionary dynamics of multidrug-resistant *S. pneumoniae* clonal complex 271 in China, revealing two globally distributed clones with distinct evolutionary histories and resistance patterns. In addition, a previous study reported a classification system for predicting β-lactam antibiotic resistance, using WGS data (Li et al., 2016).

Despite the many advantages, the analysis of WGS data requires specialized bioinformatics skills and computational infrastructure, and often involves multiple standalone software tools (Oakeson et al., 2017; Rossen et al., 2018). This complexity can make the process inaccessible to many researchers and clinicians. In recent years, several tools for analyzing pneumococcal genomes have been developed, including SeroBA, PneumoCaT, PfaSTer, and PneumoKITy (Epping et al., 2018; Kapatai et al., 2016; Lee et al., 2023; Sheppard et al., 2022). However, these analysis frameworks mainly focus on serotyping and do not integrate the examination of key features that are important in clinical practice. Although PathogenWatch (https://pathogen.watch/) offers integrated analysis functions, such as GPSC assignment and antimicrobial resistance profiling, it does not support the detection of virulence and mobile genetic elements. Therefore, there is a pressing need for an integrated, user-friendly platform that can automate the analysis of *S. pneumoniae* WGS data.

In this study, we have developed PneusPage, a function-rich and user-friendly online platform that simplifies the analysis of *S. pneumoniae* WGS data. This platform not only provides data quality check (QC), *de novo* assembly, drug-resistance prediction, and the detection of virulence and mobile genetic elements, but also enables rapid and accurate identification of MLST, core genome MLST (cgMLST), GPSC, and serotype.

## Materials and Methods

### *S. pneumoniae* WGS analysis pipeline

**Fig. 1.**
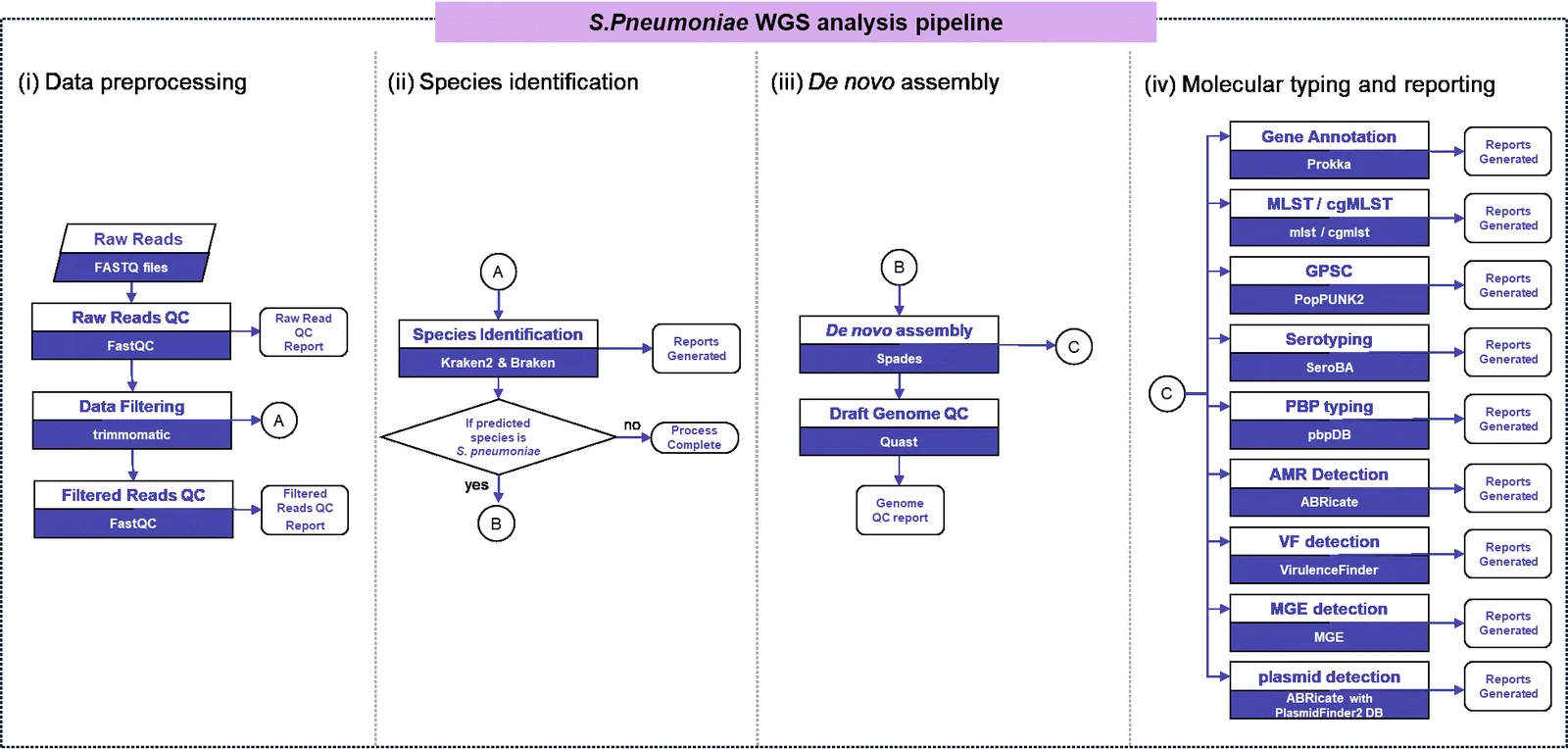
*S. pneumoniae* WGS analysis pipeline. The WGS analysis pipeline of PneusPage is divided into four main stages: (i) Data pre-processing, (ii) Species identification, (iii) De novo assembly, and (iv) Molecular typing and reporting. In the data pre-processing stage, quality control is performed on the raw sequence reads, and low-quality reads and adaptors are trimmed. During the species identification stage, the trimmed reads are analyzed to determine the species. If the strain is identified as *S. pneumoniae*, PneusPage proceeds to de novo assembly. Subsequently, clinically relevant genomic features are extracted from the trimmed sequence reads or the assembled contigs, and each result is reported accordingly. QC, quality control; MLST, multilocus sequence typing; GPSC, Global Pneumococcal Sequence Cluster; PBP, penicillin-binding protein; AMR, antimicrobial resistance; VF, virulence factor; MGE, mobile genetic element.

We developed a web-based tool named PneusPage for the genomic analysis of *S. pneumoniae*. This tool utilizes WGS data to perform analyses through four main stages: (i) Data pre-processing, (ii) Species identification, (iii) De novo assembly, and (iv) Molecular typing and reporting (Fig. 1). Through these stages, users receive detailed information on raw data quality, assembled genome quality, and assembled contigs, enabling them to thoroughly inspect sequences. During the data pre-processing stage, QC is performed on input FASTQ files using FastQC (https://www.bioinformatics.babraham.ac.uk/projects/fastqc/). Adapter recognition and trimming are performed based on the QC results using Trimmomatic (Bolger et al., 2014). Post-QC is conducted on the trimmed data, and reports are generated for all QC processes. In the species identification stage, Kraken2 (Wood et al., 2019) and Bracken (Lu et al., 2017) predict the species based on the trimmed data. If the strain is identified as *S. pneumoniae*, the sequence reads are assembled using SPAdes (Prjibelski et al., 2020). The assembled contigs are evaluated using Quast (Gurevich et al., 2013) and then used for further downstream analyses. The assemblies obtained were annotated using Prokka (Seemann, 2014). Serotypes are predicted using SeroBA (Epping et al., 2018). MLST and cgMLST are determined using the MLST tool (https://github.com/tseemann/mlst) and cgMLSTfinder (Clausen et al., 2018), respectively, with the allelic profiles from the PubMLST database (Jolley et al., 2018). Penicillin-binding protein (PBP) typing is performed using the Spn Scripts Reference (https://github.com/BenJamesMetcalf/Spn_Scripts_Reference). Acquisition of antimicrobial resistance (AMR) genes is determined using ABRicate (https://github.com/tseemann/abricate) employing the Comprehensive Antibiotic Resistance Database (Alcock et al., 2020). Virulence factors, mobile genetic elements, and plasmids are determined using VirulenceFinder (Joensen et al., 2014), MGEfinder (Johansson et al., 2021), and ABRicate with PlasmidFinder2 database (Clausen et al., 2018), respectively. GPSC is assigned through the GPS database using PopPUNK2 (Lees et al., 2019).

### Implementation of the PneusPage platform

**Fig. 2.**
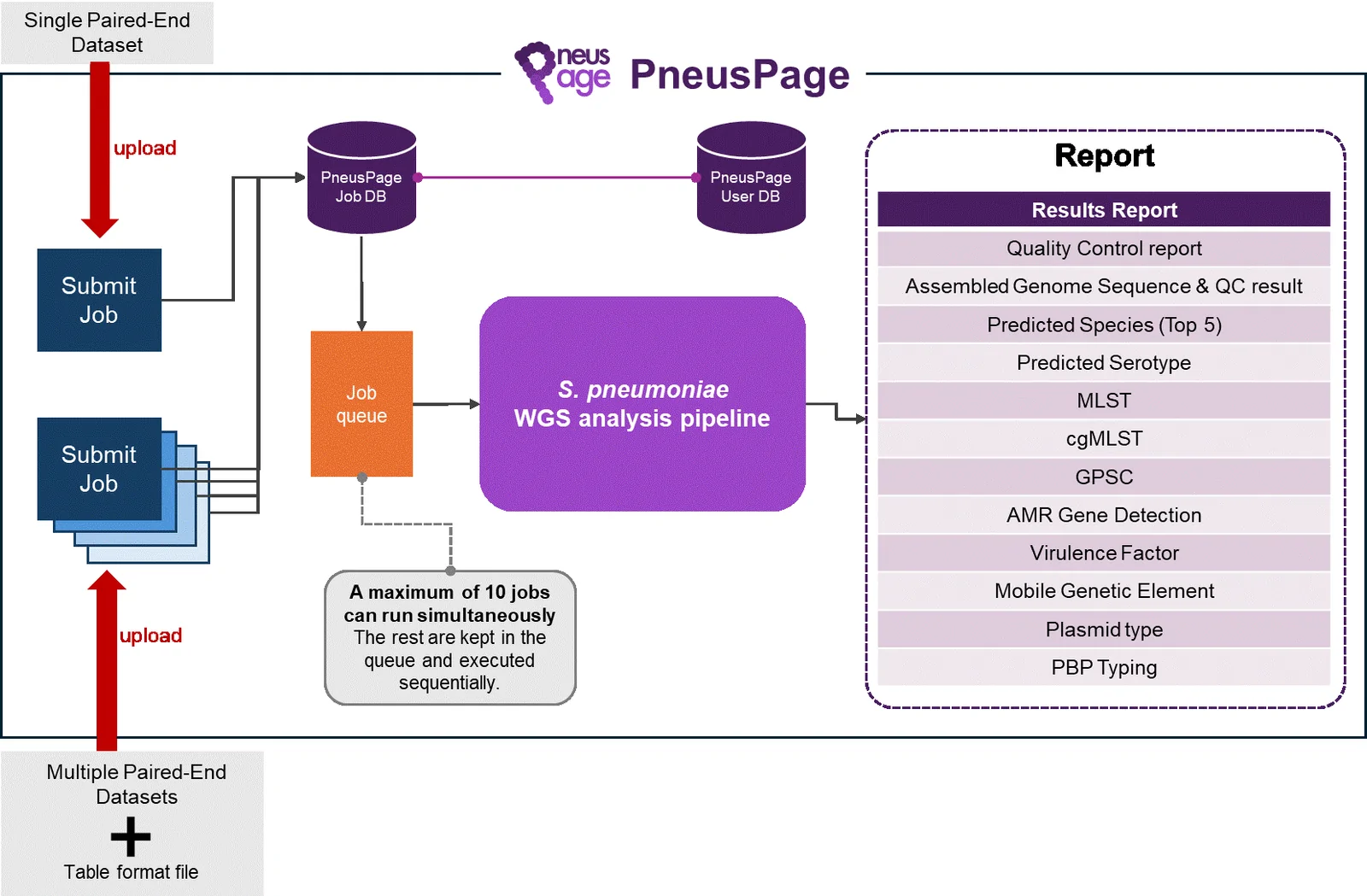
PneusPage web service structure. PneusPage consists of a database built on SQLite3, a back-end server using Flask, and a front-end UI designed with Bootstrap 5. When a user uploads raw data, the analysis begins, allowing multiple jobs to be submitted simultaneously. The server is designed to run a maximum of 10 jobs concurrently, with any additional jobs are placed in a job queue and processed sequentially. Once a job’s status is set to "running", the *S. pneumoniae* WGS analysis pipeline is executed. Upon completion, users can view a comprehensive report via the web interface. QC, quality control; MLST, multilocus sequence typing; cgMLST, core genome MLST; GPSC, Global Pneumococcal Sequence Cluster; PBP, penicillin-binding protein; AMR, antimicrobial resistance.

PneusPage is built on the Python programming language, leveraging the Flask micro web framework (https://flask.palletsprojects.com/). The web interface is constructed using HTML, CSS, and JavaScript, based on Bootstrap 5. PneusPage consists of three main pages: the “Submit” page, “Result” page, and “Detail” page. All services are accessible after logging in via Google OAuth 2.0, and user data is managed using SQLite3 (Fig. 2).

### Dataset collection for validation

We obtained 80 pneumococcal genomes with distinct serotypes from the GPS database (http://www.pneumogen.net/gps/). The raw FASTQ files for all 80 isolates were accessed through the Sequence Read Archive (SRA), with accession numbers listed in Table S1. Each isolate underwent paired-end sequencing, ensuring comprehensive coverage and accuracy in downstream analyses. To facilitate comparative analysis with PneusPage, the raw FASTQ files for all 80 isolates were also uploaded and analyzed using the PathogenWatch platform (https://pathogen.watch/).

## Results and Discussion

### Overview of PneusPage

**Fig. 3.**
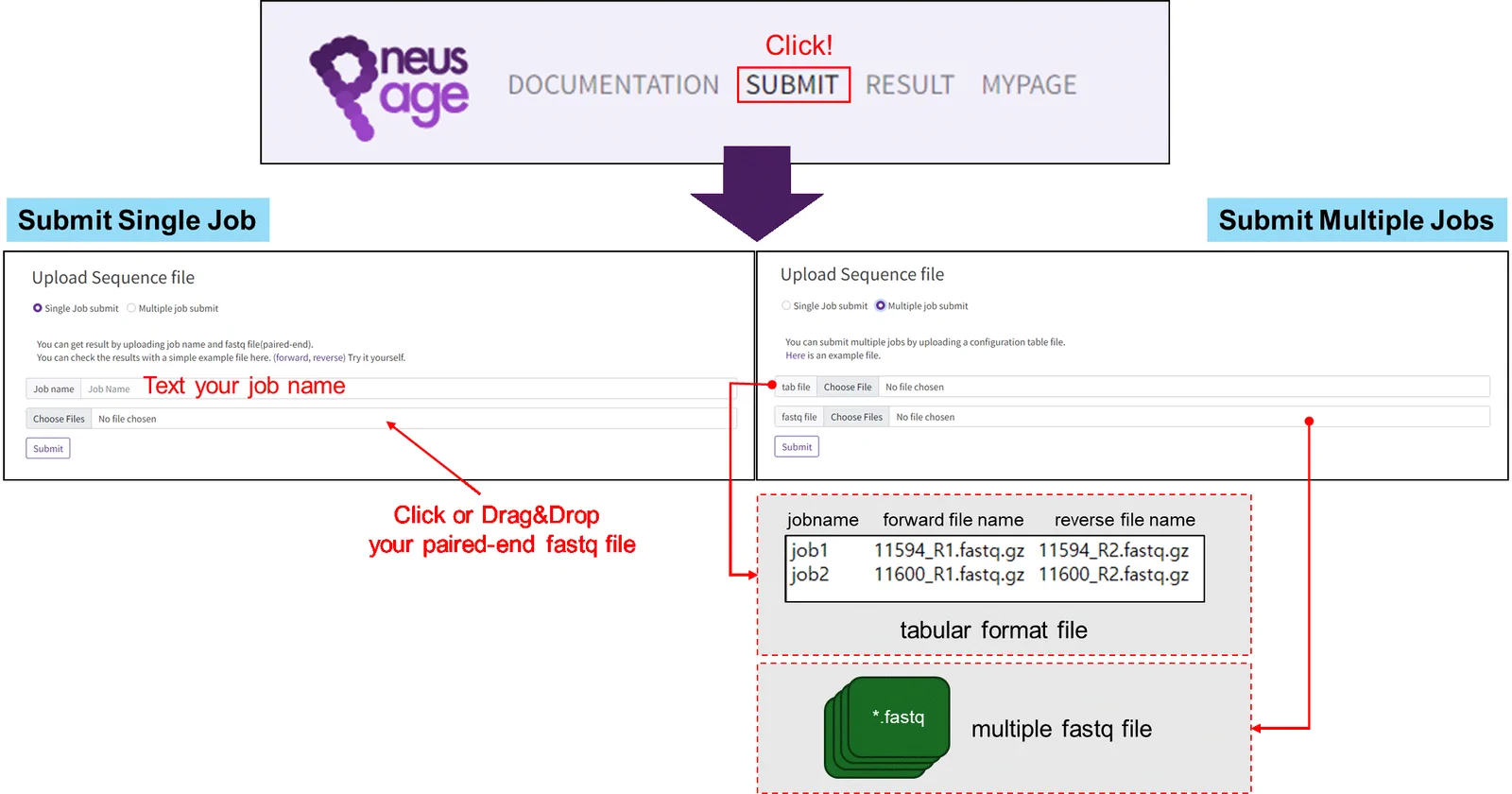
Submitting a job in PneusPage. PneusPage allows for two methods of job submission: (i) Single Job Submission and (ii) Multiple Job Submission. In the Single Job Submission method, the user provides a job name and uploads paired-end FASTQ files, which are then submitted for analysis. In the Multiple Job Submission method, the user uploads a tabular file containing the job name and forward and reverse FASTQ file names. After this, all the FASTQ files listed in the tabular file must be uploaded, and the analysis proceeds sequentially. There is no limit to the number of job submissions; however, since the server can run a maximum of 10 jobs simultaneously, any additional jobs are placed in a queue and processed when resources become available.

**Fig. 4.**
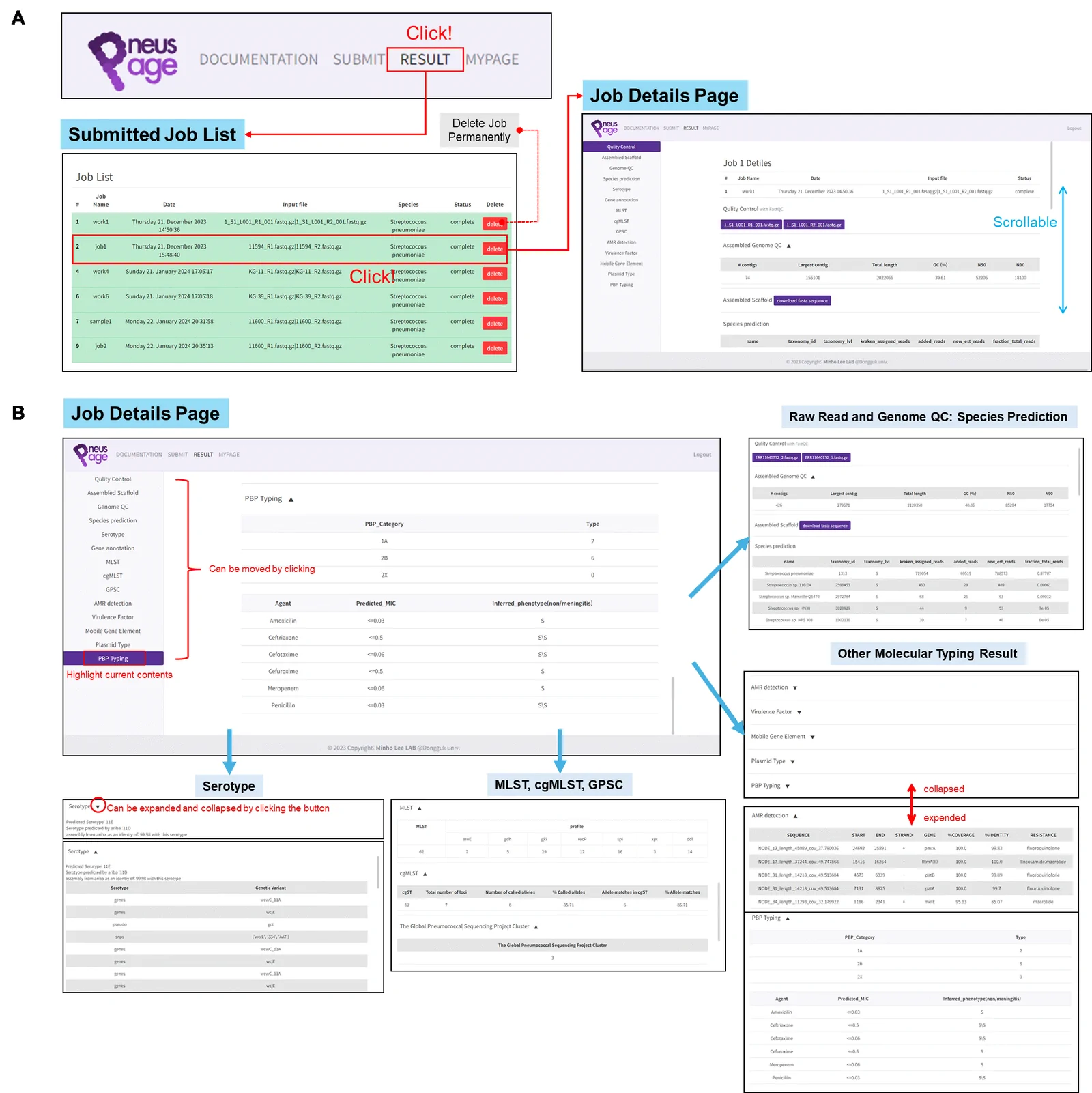
Viewing the Analysis Report in PneusPage. The analysis progress, results, and report can be viewed on the results page. The results page is divided into the submitted job list page, which allows monitoring of jobs, and the detail page, which contains specific information for each job. (A) On the Results page, users can view and manage the list of submitted jobs. When all pipelines are completed and a job's status is marked as "complete," the corresponding row turns green. These green-highlighted rows are clickable. Clicking on a row directs the user to the job's detail page. The detail page is scrollable, allowing users to view each report in sequence. Additionally, a sidebar menu enables users to directly navigate to specific sections of the report. (B) Each detail page of the results provides various information on the input sample. Users can download the quality control results of the raw data and the FASTA file for the assembled scaffold. Detailed molecular analysis results are available, including serotype, MLST, AMR detection, and virulence factors. All sections of the report can be expanded or collapsed using buttons. By default, serotype, MLST, cgMLST, and GPSC information are expanded, while AMR detection, virulence factors, mobile genetic elements, plasmid types, and PBP typing are initially collapsed.

PneusPage is a freely available web-based platform that allows users to analyze pneumococcal genomes. It accepts WGS reads in FASTQ format (two paired-end files) as input, with the option to upload multiple files for batch analysis. For a single task, users specify a job name and the FASTQ files to be analyzed (Fig. 3). For batch analysis, users submit jobs by providing a tabular text file containing job names and FASTQ file names, along with the raw FASTQ files to be analyzed (Fig. 3). Logged-in users can view information on all submitted jobs, including the current status of each job (Fig. 4A). At the end of the analysis, a report of the completed job is provided to users, and access to the detail report is only granted for jobs that have successfully executed the entire pipeline. The run time for a sample with a sequencing depth of 97-fold is approximately 25 min. PneusPage is available at https://pneuspage.minholee.net/, where users can log in with their Google accounts to upload and analyze samples. The uploaded data and processed results are retained in the user’s account, allowing the analysis results to be reviewed at any time until the user deletes them.

Users can download QC results and view the top five results for species predictions (Fig. 4B). For data identified as *S. pneumoniae*, additional information is provided, including serotype and MLST (Fig. 4B). MLST offers specific alleles and sequence types found in seven housekeeping genes (*aroE*, *gdh*, *gki*, *recP*, *spi*, *xpt*, and *ddl*), which are used to distinguish and identify the genetic diversity of *S. pneumoniae*. cgMLST analyzes multiple core genes to provide high-resolution genotyping of pneumococcal isolates, quantifying genetic similarities and differences among isolates for more precise differentiation of diverse lineages. The GPSC provides a global framework for categorizing *S. pneumoniae* strains based on their sequence data, facilitating the understanding of the distribution and evolution of different clones worldwide and helping to track the spread of pathogenic and resistant strains. All of these lineage distinction results (MLST, cgMLST, and GPSC) are highly useful for epidemiological surveillance and infection control (Belman et al., 2024; Spanelova et al., 2020).

PneusPage further provides an AMR detection report, which includes sequence coverage and identity, a list of AMR genes, and predictions of phenotypic resistance to various antibiotics (Fig. 4B). In addition to AMR detection, PneusPage performs PBP typing based on mutations in PBPs (PBP1a, PBP2b, and PBP2x), predicting the resistance phenotype to β-Lactam antibiotics (Li et al., 2016). Both AMR detection and PBP typing can assist clinicians in determining treatment options. Finally, PneusPage offers functions for identifying MGE, virulence factor, and plasmids (Fig. 4B). The MGE detection function focuses on identifying elements such as transposons, integrons, and prophages that facilitate the horizontal transfer of genes, including those related to antibiotic resistance and virulence factors. The results allow for the identification of the MGE list, the types of each MGE, and the sequence coverage and identity with reference sequences. The Virulence Factor detection function identifies genes associated with virulence, providing results such as the specific virulence factor detected, sequence identity, contig information, and predicted virulence protein function. The Plasmid Type function provides rapid detection of known plasmid types based on replicon sequences. The detection of MGE, virulence factor, and plasmid is helpful in identifying specific virulence factors associated with phenotypes and understanding the mechanisms of horizontal gene transfer.

### Validation of PneusPage

To verify the accuracy of PneusPage, we collected 80 pneumococcal WGS datasets with different serotypes from the GPS project and compared the results from PneusPage with those obtained from the PathogenWatch platform. Regarding serotypes, the concordance between the metadata provided by the GPS project and the results from PneusPage was 97.5%, which was 2.5% higher than that of PathogenWatch (Table 1). Two isolates reported as serotypes 33A were correctly predicted by PneusPage, while PathogenWatch inconsistently predicted them as 33F, respectively (Table S1). This suggests that subclassification within the same serotype may be difficult due to sequence similarity in the capsular polysaccharide (Elberse et al., 2011). Two other isolates reported as serotypes 12A and 12B were predicted by both PneusPage and PathogenWatch to be serotypes 46 and 12F, respectively, suggesting uncertainty in the metadata for these samples (Table S1).

We also performed a comparative analysis of MLST and GPSC. The MLST analysis showed agreement between the GPS project metadata, PathogenWatch, and PneusPage for all seven housekeeping genes. As a result, the sequence type of each isolate predicted by both platforms was 100% consistent with the GPS project metadata (Table 1 and Table S2). In the GPSC analysis, the concordance between the metadata provided by the GPS project and the results from PneusPage was 100%, while PathogenWatch showed a concordance of 98.8% (Table 1). The discrepancy in PathogenWatch was due to unassigned isolates, which were assigned as GPSC16 in both the metadata and PneusPage, respectively (Table S3). Regarding PBP typing, the concordance between the metadata and PneusPage for PBP1a, PBP2b, and PBP2x was 91.3%, 95.0%, and 95.0%, respectively, while PathogenWatch showed concordance of 98.8%, 100%, and 100%, respectively (Table 1 and Table S4). Taken together, the genome analysis pipeline implemented by PneusPage provides more accurate predictions in terms of serotype and GPSC analysis compared to the PathogenWatch platform.

PneusPage stands out from other genome analysis services by providing a wealth of information simply by uploading data. In addition to offering data QC and molecular typing, PneusPage provides draft genome, allowing users to examine the sequences themselves. The biggest differentiator from similar services like PathogenWatch is the analysis of additional information, such as virulence factors, mobile genetic elements, and gene annotations, which provide a basis for studying the mechanisms of AMR gene spread or identifying markers for invasive pneumococcal diseases. PneusPage also supports batch analysis, enabling users to run up to 10 jobs simultaneously, thereby increasing the efficiency of genomic analysis. However, this study also has some limitations. First, PneusPage has only been validated on paired-end sequencing data generated by Illumina platforms. Second, although there is no limit on data upload size, running 10 jobs simultaneously may not be sufficient for population-level analyses. Future updates and improvements to PneusPage are needed to accept data from various sequencing platforms and to increase the number of simultaneous tasks. Third, the PBP typing agreement of PneusPage was slightly lower than that of PathogenWatch, which may have been influenced by pre-processing of the WGS data or differences in PBP typing databases between the two tools. Specifically, different parameters for low-quality base trimming and *de novo* assembly can result in discrepancies in the draft genomes produced from the same input data, which also affects PBP typing. Additionally, PneusPage implemented the machine learning model and database developed by Li et al. (2016), who first developed the WGS-based PBP typing system, whereas it is unclear whether PBP typing in PathogenWatch uses the same database as our software for PBP typing. Further improvement of the machine learning models based on large dataset with phenotypic drug susceptibility testing, as well as regular updates to PneusPage’s database, will be useful for monitoring antimicrobial resistance.

In summary, PneusPage offers a rapid and efficient platform for analyzing WGS data, accurately predicting *S. pneumoniae* as well as its resistance and molecular profiles. By simplifying the interpretation of pneumococcal WGS data, PneusPage could play a key role in strengthening global efforts to combat this infectious disease and aid in the development of novel vaccine strategies.

## Figures and Tables

**Table 1.** Comparison of Concordance Rates for Serotype, MLST, GPSC, and PBP typing Across Different Analysis Platforms

Category	Subcategory	GPS - PneusPage	GPS - PathogenWatch	PneusPage -PathogenWatch
Serotype		97.5% (78/80)	95%(77/80)	100% (80/80)
MLST	Sequence Type	100% (80/80)	100% (80/80)	100% (80/80)
	aroE	100% (80/80)	100% (80/80)	100% (80/80)
	gdh	100% (80/80)	100% (80/80)	100% (80/80)
	gki	100% (80/80)	100% (80/80)	100% (80/80)
	recP	100% (80/80)	100% (80/80)	100% (80/80)
	spi	100% (80/80)	100% (80/80)	100% (80/80)
	xpt	100% (80/80)	100% (80/80)	100% (80/80)
	ddl	100% (80/80)	100% (80/80)	100% (80/80)
GPSC		100% (80/80)	98.75% (79/80)	98.75% (79/80)
PBP-Typing	pbp1a	91.25% (73/80)	98.75% (79/80)	92.5% (74/80)
	pbp2b	95% (76/80)	100% (80/80)	95% (76/80)
	Pbp2x	95% (76/80)	100% (80/80)	95% (76/80)
	pbp1a;pbp2b;pbp2x	88.75% (71/80)	98.75% (79/80)	88.75% (71/80)

This table presents the concordance rates between the GPS project database samples and two analysis platform, PathogenWatch and PneusPage, across four key *S. pneumoniae* typing categories: Serotype, MLST, GPSC, and PBP Typing. The MLST is divided into seven housekeeping genes and sequence type. The PBP typing is subdivided into three specific penicillin-binding proteins and their combination. Concordance percentages are shown between the GPS project dataset and each tools, as well as between the PathogenWatch and PneusPage.

## Supplementary Information

Table S1. Comparison of serotypes between the GPS project, PathogenWatch, and PneusPage.

Table S2. Comparison of MLST between the GPS project, PathogenWatch, and PneusPage.

Table S3. Comparison of GPSC between the GPS project, PathogenWatch, and PneusPage.

Table S4. Comparison of PBP typing between the GPS project, PathogenWatch, and PneusPage.
